# Editorial: The Physiology and Pharmacology of Leucine-rich Repeat GPCRs

**DOI:** 10.3389/fendo.2016.00056

**Published:** 2016-06-17

**Authors:** Brian J. Arey, James A. Dias

**Affiliations:** ^1^Research and Development Bristol-Myers Squibb Co., Princeton, NJ, USA; ^2^University at Albany, State University of New York, Albany, NY, USA

**Keywords:** G protein-coupled receptors, leucine-rich repeat family of receptors, LGR, GPCRs, physiology and pharmacology

**The Editorial on the Research Topic**

**The Physiology and Pharmacology of Leucine-rich Repeat GPCRs**

G protein-coupled receptors represent a large family of proteins that act as receptors for many types of physiological ligands, including peptides, metabolites, and lipids. These receptors are important for understanding physiology since they contribute to the regulation of all major organ systems. Additionally, they are also a key focus for the development of therapeutics for the treatment of pathophysiology and are still recognized as the most druggable class of macromolecules today. GPCRs are classified into separate subfamilies (Classes A, B, and C) based on protein sequence homology in their transmembrane domains. Within the Class A family of GPCRs, these receptors can be further placed into sub-groups based on other structural features and similarities in function. In this Special Topic for Frontiers in Endocrinology: Molecular and Structural Endocrinology, we have focused on a subfamily of Class A GPCRs, the leucine-rich repeat family of receptors (LGR). The Physiology and Pharmacology of Leucine-rich Repeat GPCRs captures the continuum of structure to function, agonist to effector, and reproduction to metabolism that provides an overview of this important family of receptors.

The LGRs are characterized by the leucine-rich repeat structural motif (1) that provides the rigid structure of their large extracellular domains. The predicted heptahelical transmembrane domains and their sequence homology in this region with other receptors classify them as Class A GPCRs (2). Furthermore, there are currently three sub-groups of LGRs recognized. The Type A receptors have been extensively studied and are receptors for the pituitary and placental glycoprotein hormones. The endogenous ligands for the Type B (R-spondins) and C (relaxin) receptors have only recently been identified and rapid progress has been made that has advanced understanding of their structure and function. These receptors are important mediators in the regulation of diverse physiological process, such as reproduction, cardio-renal function, cell growth, and stem cell differentiation. Within this Special Topic, we seek to provide an understanding of this family of receptors while addressing both future opportunities and challenges that lay ahead.

Petrie et al. provide an overview of LGRs in terms of structure and organization that places this family of receptors within the larger context. This review provides in depth knowledge of the RXFP1 and RXFP2 receptors whose cognate ligands are two insulin- related peptides, H2 relaxin, and INSL3. Thus, this review while calling out the uniqueness of the Type C LGRs RXFP1 and RXFP2, also introduces us nicely to the three Types of LGRs based on the size of their leucine-rich repeat extracellular domains.

Continuing on this theme of structure and function, activation of the most well studied of the LGRs, the gonadotropin receptors, is reviewed. The structural basis of activation of the gonadotropin receptors in the absence of a full crystal structure remains an enigma. However, we do know that the large extracellular domains are the binding site of the large heterodimeric ligands for these receptors. A remaining question is the potential role of the other parts of the receptor structure in determining function. Banerjee and Mahale provide evidence using site-directed mutagenesis that signaling of the LH receptor is dependent on specific residues of the extracellular loops. Furthermore, Grzesik et al. demonstrate that differences in signaling by the two physiological ligands of the LH receptor, are at least in part, mediated by the hinge region of the extracellular domain. While both receptors coexist in the same cell during folliculogenesis, it is unclear how their agonist-induced signals are parsed out. Further refining this thought, this paper addresses how one receptor can bind two nearly identical ligands and produce two different signaling profiles. It turns out that both hormone and extracellular loops coordinate to produce the breadth of nuance seen in signaling of these receptors. This seems a critical point if small molecules are to be developed which mimic some or all of these signaling patterns.

The physiological and therapeutic importance of the Type B and C receptors is discussed as well. The importance of RXFP1 in cancers is exemplified by Thanasupawat et al. in a review of new ligands for RXFP1 other than the canonical relaxin and INSL3; specifically C1q/TNF-related proteins and the role of RXFP1 as a brain cancer promoter. The theme is further explored with an in-depth look by Li et al. of the Type B LGR4 where we learn that this receptor that is critical for developmental signaling and tissue homeostasis has as its ligand, R-spondins. We learn that R-spondins are the sole secreted potentiators of Wnt signaling and stem cell maintenance and appreciate that the Type B LGRs do not signal via G-proteins but do interact with the FZD–LRP complex to stimulate unique signaling pathways.

The LGR receptor family has been the subject of drug development for decades. Focusing on improving the natural ligand for therapeutics based on these receptors, a perspective is provided by Szkudlinski on a journey toward the successful development of superagonists of the thyroid-stimulating hormone receptor, a Type A LGR. This review shines light on the potential of utilizing the naturally occurring ligands as a scaffold for engineering by structure-based drug design to develop “super biosimilars.” This is contrasted with the development of small molecule agonists and antagonists that act at this same class of LGR Type A as described by Nataraja et al. Here, we learn that it is possible to bypass the complicated interactions of heterodimeric ligands and the large extracellular domains of the LGR Type A receptors, to effect activation or inhibition with molecules a fraction of the size of the natural ligands. Although this represents a clear step forward in our ability to develop small molecule agonists and antagonists to these receptors, it is not without its challenges. In what would seem to be a reasonable assumption that cAMP as readout would predict efficacy *in vivo*, this is shown to not necessarily be the case. In the end, primary cells and iterative testing reveal the true candidate.

This concept is further exemplified in the article by Huang et al. An overwhelming majority of the preclinical animal testing for relaxin treatment includes rodent models and, thus, the inability of small molecule agonists to activate the mouse receptor has hampered preclinical studies. In a search for animal models to study RXFP1 small molecule agonists as potential acute heart failure therapeutics, it was determined that non-human primate and porcine species could be used but the standard laboratory mouse model was unable to respond to the lead compound! These examples illustrate how development of small molecule therapeutics is fraught with potential pitfalls and how appropriate models are needed for screening and selection of leads.

Finally, in an era of optogenetics and real-time inquiry, the use of transgenic methods may yield some recourse. Narayan describes how, for the gonadotropin receptors, a combination of knockout and knock-in approaches can yield novel mouse models that either simulates human disease or tests whether genomic variants can explain disease. In this regard, the luteinizing hormone receptor has been very well studied using transgenic animals to better understand the effect of mutations causing constitutive activation as a model for Familial Male Precocious Puberty.

Advanced methods in cellular imaging are also available which will aid in the study of LGR signaling. Certainly in the area of the gonadotropin receptors, these methods have contributed to an understanding that, although the canonical cAMP pathway is operative in the gonadotropin receptors, additional pathways are likely at play. Thus, Ayoub et al. describes the use of bioluminescence resonance energy transfer (BRET) to study activation of gonadotropin receptors in living cells. Using this method, they confirm that these receptors exhibit biased agonism.

It has been nearly a century since Smith discovered the relationship between pituitary extracts and follicular development and 60 years since Hisaw discovered relaxin as a hormone that could cause a loosening of the pubic symphysis prior to parturition. These seminal findings have led to the identification of the importance of these peptides on the physiology of the reproductive system but have also ultimately revealed a more complex endocrine role of these hormones and the identification of a unique family of receptors. Research over the ensuing decades has revealed that the LGRs exert varied and comprehensive controls on processes that include but are certainly not limited to reproduction and point to their potential as therapeutic targets to treat disease.

## Author Contributions

BA and JD contributed equally to this manuscript.

## Conflict of Interest Statement

The authors declare that the research was conducted in the absence of any commercial or financial relationships that could be construed as a potential conflict of interest.
